# Analysis of the access of pregnant women to the first programmatic dental appointment: an ecological study

**DOI:** 10.1590/1807-3107bor-2024.vol38.0012

**Published:** 2024-01-05

**Authors:** Camila MARIOTTI, Leiriane Alves de SOUZA, Luiz Renato PARANHOS, Jaqueline Vilela BULGARELI, Álex Moreira HERVAL

**Affiliations:** (a) Universidade Federal de Uberlândia – UFU, School of Dentistry, Department of Community and Preventive Dentistry, Uberlândia, MG, Brazil.

**Keywords:** Maternal Health, Health Care Disparities, Public Health, Primary Health Care, Socioeconomic Factors

## Abstract

To plan and evaluate public health policies, it is important to understand the influence of social factors on the quality and access to dental care. This study aimed to verify the potential association between the indicators of pregnant women receiving dental care and the social and health care indicators of cities in the Brazilian state of Minas Gerais. A cross-sectional ecological study was performed with secondary data from the Brazilian Institute of Geography and Statistics and the Health Care Department of the Ministry of Health regarding the cities of Minas Gerais. The study analyzed three health care indicators (such as more than six prenatal, the proportion of syphilis and human immunodeficiency virus tests, and oral health coverage) and four social indicators (average monthly wage, illiteracy rate, proportion of employed population, and rate of adequate sanitary sewerage). Bivariate analysis (Mann-Whitney test) and logistic regression were performed using Jamovi software. All of the indicators analyzed were associated with the access of pregnant women to dental care. However, in the regression models, only health care indicators remained statistically significant. Thus, although social indicators are associated with the access of pregnant women to dental appointments, access to primary health care and the teamwork of primary health care teams may overcome social inequality in the access of pregnant women to dental care.

## Introduction

Different policies directed toward maternal and child health have been implemented in Brazil to ensure quality health care for pregnant women and children.^1,2^ The care of pregnant women is incorporated into the Brazilian Oral Health Policy, which includes collective actions of health promotion and prevention as well as the performance of curative procedures when required.^3^

Some indicators have been used to monitor these policies and to subsidize the planning, formulation, and implementation of new public health policies.^4-6^ Three indicators are currently used in Brazil to assess the care of pregnant women: the proportion of pregnant women with: a) at least six prenatal appointments performed up to the 20^th^ week of pregnancy; b) syphilis and human immunodeficiency virus tests performed; and c) dental care performed .^7^

The oral health indicator assesses the percentage of pregnant women who have access to oral health care in the primary health care system to create a preventive, educational, and therapeutic care plan.^7,8^ This indicator is significant in the context of public health because it reflects the expansion and consolidation of oral health teams,^9^ as well as the potential of services to provide integral care to the mother-child binomial.^10^ However, it must be considered that access to health care is not only influenced by the expansion and use of health services^11^ but also by socioeconomic variables such as living conditions,^12^ level of education, economic class, marital status, and lifestyle.^13^ When accepting the social health determinants in the analysis of access to health care, it should be understood that social inequities aggravate the inequality of access to oral health care.^8,11^

Formulating policies that seek to extend the coverage of oral health care requires understanding the factors that influence access to health care.^11^ Thus, this study aimed to analyze the association of pregnant women’s access to dental appointments with social and health care indicators in cities in the Brazilian state of Minas Gerais. This study presents the initial null hypothesis that social and health care indicators are not related to pregnant women’s access to their first programmatic dental appointment.

## Methodology

### Study design and protocol

A cross-sectional ecological study was performed based on the social and health care indicators disclosed by government agencies. The research did not require project submission to the Research Ethics Committee because it used secondary data from the public domain offered by the Ministry of Health and the Brazilian Institute of Geography and Statistics (IBGE), and it agreed with Resolution No. 510 of April 7, 2016 of the Brazilian Health Council. The Strengthening the Reporting for Observational Studies in Epidemiology tool was used to organize the research and publish the results.

### Study context and variables

This study was performed in the Brazilian state of Minas Gerais and considered all 853 cities, with a total population of 19,597,330 inhabitants. It is estimated that the state has approximately 274,007 pregnant women annually. The access of pregnant women to their first programmatic dental appointment and, consequently, the health care target for pregnant women, is a public policy stimulated by state and federal governments. At the federal level, this indicator was integrated into the Prevention Brazil program, representing the only goal for oral health teams in Brazil. At the state level, the Stork Network program was created to ensure higher accessibility to maternal and child care services, including oral health care. Thus, access for pregnant women to their first programmatic dental appointment is a priority for oral health teams, the focus of which has remained during the coronavirus disease 2019 pandemic. At this crucial time, oral health teams were instructed to maintain the indicator goal, reorganize the care provided to pregnant women on the same day as other appointments for pregnant women, introduce non-face-to-face monitoring (teledentistry), develop urgent and emergency care, and maintain educational and preventive activities.

Data on health care indicators (oral and maternal-child) were collected considering the year 2020. Regarding social indicators, the most recent data offered by the IBGE were used. The studied variables were the health and social indicators available in the Ministry of Health and IBGE databases. The social indicators included: a) income (average monthly wage, which refers to the average wage of formal city employees); b) level of education (illiteracy rate, which indicates the number of people over the age of 15 who cannot read and write the language in comparison to the total population of the city); c) occupation (proportion of the employed population, which indicates the proportion of the population that performs a professional activity); and d) urban infrastructure (rate of adequate sanitary sewerage, which indicates the number of people with a waste sink in their residence connected to the waste collection system or a septic tank). The health care indicators collected were: the proportion of pregnant women a) with at least six prenatal appointments performed, with the first visit up to the 20^th^ week of pregnancy; b) with syphilis and human immunodeficiency virus tests performed; c) with oral health coverage; and d) who received dental care. All indicators collected were continuous numeric values.

### Data collection

The data on social indicators (average monthly wage, illiteracy rate, proportion of employed population, and rate of adequate sanitary sewerage) were obtained from the IBGE website (https://cidades.ibge.gov.br). The data on health care indicators were collected with the Panel of Indicators of the Primary Health Care Department of the Ministry of Health (SAPS/MS) in a field that provides detailed information by city (https://sisaps.saude.gov.br/painelsaps/situacao-geral). Additionally, data on the coverage of dental services were collected from the Portal of Basic Care Information and Management (e-Gestor AB/MS) (https://egestorab.saude.gov.br).

### Data analysis

The data were tabulated in a Microsoft Excel spreadsheet. For statistical analysis, the tabulated data were imported into the Jamovi™ software. Initially, the data were descriptively analyzed by calculating the measures of central tendency and data dispersion. The median value, which was 15.7, was the cutoff point for dichotomizing the outcome variable (first programmatic dental appointment). Thus, municipalities with access to the first programmatic dental appointment <15.7 were classified as having the lowest access to the first programmatic dental appointment, and those with values ≥15.7 represented higher access. The Kolmogorov-Smirnov and the Levene tests were used to verify the normality assumptions and homogeneity of variances, respectively, to determine the statistical analysis that best fits the data. Thus, the Mann-Whitney U test was used to determine the association of social and health care indicators with access to the first programmatic dental appointment. Finally, the data were analyzed in a multivariate manner using logistic regression. Before inserting the data into the regression, the collinearity of variables was tested (variable inflation factor). To produce the regression model, the health care indicators (variables), followed by the social indicators, were included.

## Results

The social and health care indicators of 853 cities in the Brazilian state of Minas Gerais were analyzed. Table 1 presents the bivariate analysis of the association between social and health care indicators and access to the first dental appointment. All of the social and health care indicators that were analyzed showed a statistically significant association with access to the first programmatic dental appointment.

**Table 1:** Distribution of the cities according to the social and health care indicators analyzed and the association with access to the first programmatic dental appointment.

Indicators analyzed	Access to the programmatic dental appointment		

	Lowest access	Highest access	p-value

	Mean ± SD	Mean ± SD	
Oral health coverage	81.7 ± 27.8	94.0 ± 15.1	> 0.001
Pregnant women with more than six prenatal appointments	15.8 ± 17.6	38.1 ± 20.0	> 0.001
Pregnant women with STI tests	15.1 ± 16.2	34.7 ± 20.8	> 0.001
Income	1.85 ± 0.45	1.77 ± 0.39	0.005
Level of education	12.3 ± 6.18	14.6 ± 6.52	> 0.001
Urban infrastructure	62.9 ± 24.3	55.3 ± 26.5	> 0.001
Occupation	16.2 ± 9.37	14.9 ± 8.90	0.003

SD: standard deviation; STI: sexually transmitted infection; p < 0.05

Table 2 presents the results of the multivariate analysis (binomial logistic regression), which were inserted in three steps. In the first model, only access to oral health was considered for analysis because it is an essential element for the access of pregnant women to their first programmatic dental appointment (R^2^ = 0.0547). In the second model, the other two health care indicators were added, which referred to the access of pregnant women to health care (pregnant women with more than six prenatal appointments and who had sexually transmitted infection tests). The inclusion of these indicators promoted an important increment in the explanatory power of the model (R^2^ = 0.2920). The third model included all the social indicators studied. There was a discrete increase in the explanatory power of the model (R^2^ = 0.2935). However, the social indicators were not statistically significant. The statistical comparison model (not shown in the tables) indicated a statistically significant difference between Models 1 and 2 (p < 0.001). However, there was no statistically significant difference between Models 2 and 3 (p = 0.771).

**Table 2:** Multivariate analysis of the prediction of social and health care indicators on the access of pregnant women to the first programmatic dental appointment.

Indicators analyzed	Access to the programmatic dental appointment					

	Model 1		Model 2		Model 3	

	(R^2^ = 0.0547)		(R^2^ = 0.2920)		(R^2^ = 0.2935)	

	OR1 (95%CI)	p-value	OR2 (95%CI)	p-value	OR3 (95%CI)	p-value
Oral health coverage	1.027	> 0.001	1,029	> 0.001	1.027	> 0.001
	(1.019–1.035)		(1.020–1.037)		(1.018–1.036)	
Pregnant women with more than six prenatal appointments			1,042	> 0.001	1.042	> 0.001
			(1.032–1.052)		(1.031–1.052)	
Pregnant women with STI tests			1,036	> 0.001	1.036	> 0.001
			(1.025–1.047)		(1.025–1.047)	
Income					1.024	0.924
					(0.624–1.681)	
Level of education					1.019	0.299
					(0.983–1.056)	
Urban infrastructure					0.998	0.745
					(0.990–1.006)	
Occupation					1.007	0.568
					(0.982–1.033)	

CI: confidence interval; STI: sexually transmitted infection; OR: odds ratio; p < 0.05

## Discussion

The ecological analysis of the indicators of the cities in the Brazilian state of Minas Gerais showed that both the social and health care indicators analyzed were associated with the access of pregnant women to their first programmatic dental appointment. However, in the multivariate analysis, only health care indicators remained statistically significant. The non-statistically significant difference observed in Model 3, which includes health and social indicators, may indicate that the work of primary health care teams may overcome the social inequality in the access of pregnant women to dental care.

Understanding the factors that influence access to dental care is essential for developing health policies at various management levels to achieve universal access to oral health. Working on access to health care includes individual and interpersonal factors, characteristics of health professionals and health services, and organizational, political, and social factors.^14^ Patient’s lifestyle, schedule flexibility, technological skills,^14^ presence or absence of a family and social support network,^11,14^ and type of health care demand perceived by patients should be considered at the individual level.^11^ Communication barriers between professionals and patients, as well as the lack of a trusting relationship, may act as barriers to access.^15,16^ Political factors, such as the public health funding model, shortage of resources, and lack of workers, should also be considered. Social and cultural factors related to culture, family, and health beliefs and stigma may also strongly affect individuals’ access to health care.^17^

Regarding the access of pregnant women to health care, long distances between residences and health care units should be considered because they imply time and money spent, complicating their access to dental appointments.^18^.These physical barriers are associated with myths, beliefs, and insecurities in pregnant women regarding the performance of safe dental treatment during pregnancy.^10,19,20^ Thus, resistance to dental treatment overlaps with the scientific evidence that shows the benefits and low risks of dental treatment during pregnancy.^21,22^

The present study emphasizes the importance of social factors in determining access to health care, as the socioeconomic characteristics of the cities analyzed were associated with the access of pregnant women to their first programmatic dental appointment. Consistent with this result, socioeconomic inequalities are associated with access to and use of dental services,^23^ and low-income pregnant women residing in smaller cities with lower human development indices receive fewer prenatal instructions.^24^ The analysis of the federal units of Brazil also showed a negative correlation between the human development index and access to dental care.^8^ Hence, the implications of social inequalities on access to adequate health care should be considered.

It took decades to acknowledge the influence of social health determinants, so this paradigm would gradually be consolidated. When understanding the influence of social characteristics on human health, it becomes indispensable to address social inequalities to improve the living conditions of the population and the distribution of income and power.^25^ As a result, concrete strategies with the true potential to mobilize society, produce change, and promote health equity should be developed. ^25^

Social inequality in access to oral health care indicates that the Brazilian health system still fails to ensure universal and equal access.^23^ Despite these challenges, strategies that seek to reduce the impact of social inequalities on the access and coverage of health care have been increasingly improving health care indicators in the care of pregnant women^26^ and access to dental care.^8^ Prioritizing equitable policies for pregnant women and newborns is indispensable for fighting inequalities in access to health care. Therefore, the integral care of pregnant women should be based on humanized and qualified care, considering the social health determinants of each pregnant woman.^27,28^ These findings highlight the need for new public policies that consider pregnant women’s living conditions and the part of society to which they are contributing, to improve women’s access to health care and ensure an integrated approach to care.^29^

In the multivariate models proposed in the present study, only the indicators related to health care remained related to pregnant women’s access to their first programmatic dental appointment. This result may indicate that expansion policies for health care have decreased inequity in access to health care. In this sense, the analysis performed in Brazilian federal units showed a pro-equitable tendency in access to primary oral health care.^8^ The results of the multivariate analysis also reinforce the importance of expanding and integrating oral health teams with other primary health care professionals.^22^ According to a study conducted in the Brazilian state of Pernambuco, the health care organization model is also related to access to the first programmatic dental appointment.^30^ It should be noted, however, that several health teams adhere to the traditional care model, which focuses on individualized curative actions, limiting the potential for changing health care indicators.^6^

The indicator of access to the first programmatic dental appointment allows assessment of the tendency for expansion of the Brazilian Oral Health Policy regarding the coverage of oral health care in primary health care.^9^ Corroborating this rationale, the results of the present study indicated a consistent association between access to oral health care in primary health care and their first programmatic dental appointment. A positive association between the two indicators was observed by Raimundo et al.^31^ It is worth noting that because of the current political and economic scenario in Brazil, there has been a decrease in oral health coverage, which may be due to the low investments in the field.^32^

The results of this study are not free of limitations. One of these limitations is that some of the indicators used in the study were based on data directly provided by the cities to the information systems of the Ministry of Health, which may imply original registration failures. Another limitation is related to the characterization data of the cities collected by IBGE, which are presented as estimates and may not reflect the reality of the cities in the same period as the health care indicators.

## Conclusion

The study showed that social indicators of income, level of education, occupation, and urban infrastructure are related to the access of pregnant women to their first programmatic dental appointment, reaffirming the importance of social health determinants for planning access to oral health care. However, only the health care indicators were related to the access of pregnant women to oral health care in the multivariate analysis, indicating that implementing primary health care may reduce social inequality in access to oral health. This result also indicates the importance of continuing to expand oral health teams in primary health care and the need for effective integration among professionals working in these services.
